# Promoting knowledge to policy translation for urban health using community-based system dynamics in Brazil

**DOI:** 10.1186/s12961-020-00663-0

**Published:** 2021-04-01

**Authors:** Lidia Maria de Oliveira Morais, Jill Kuhlberg, Ellis Ballard, Katherine Indvik, Solimar Carnavalli Rocha, Denise Marques Sales, Letícia de Oliveira Cardoso, Nelson Gouveia, Amélia Augusta de Lima Friche, Waleska Teixeira Caiaffa

**Affiliations:** 1 Observatory for Urban Health in Belo Horizonte, Federal University of Minas Gerais, Av. Pres. Antônio Carlos, 6627 - Pampulha, Belo Horizonte, MG 31270-901 Brazil; 2 System Stars, St. Louis, MO United States of America; 3 Social System Design Lab, Brown School At Washington University in St. Louis, 1 Brookings Drive, St. Louis, MO 63130 United States of America; 4 Dornsife School of Public Health, Urban Health Collaborative, Drexel University, 3215 Market Street, Philadelphia, PA 19104 United States of America; 5 Oswaldo Cruz Foundation - National School of Public Health, Rio de Janeiro, R Leopoldo Bulhoes, 1480, room 813, Manguinhos, Rio de Janeiro, CEP 21041-210 Brazil; 6 Department of Preventive Medicine, University of São Paulo Medical School, Brazil. Av. Dr. Arnaldo, 455, São PauloCerqueira César, CEP 01246903 Brazil

**Keywords:** Knowledge to policy translation, Community-based system dynamics, Urban health, Framework method, Latin America, Systems thinking

## Abstract

**Background:**

Effectively bridging the knowledge–policy gap to support the development of evidence-based policies that promote health and well-being remains a challenge for both the research and policy communities. Community-based system dynamics (CBSD) is a participatory modelling approach that aims to build stakeholders’ capacity to learn and address complex problems collaboratively. However, limited evidence is available about the contributions of CBSD to knowledge-generating and policy processes across sectors and policy spheres. In the context of a multi-country research project focused on creating an evidence base to inform urban health policies across Latin America, a series of CBSD workshops convened stakeholders from research, policy-making, and other backgrounds working in food and transportation systems. Diverse participants were selected aiming to incorporate multiple perspectives relevant to understanding complex urban systems linked to food and transportation. This study focuses on one of these workshops, whose avenue was São Paulo, Brazil, assembling country-based participants representing local, regional, national, and international institutions with multidisciplinary backgrounds linked to food and transportation systems.

**Objective:**

The aim of this case study is to explore the perceived influence of one of these workshops on attendees’ understandings of food and transportation systems and their relationship to healthy urban environments, with attention to the role of the workshop in supporting knowledge to policy translation for urban health.

**Methods:**

We conducted 18 semi-structured qualitative interviews with attendees one year after their participation in a CBSD workshop held in São Paulo, Brazil. A framework method approach was used to code participants’ responses and identify emerging themes.

**Results:**

Participants reported that the workshop’s group model-building activities influenced their understanding of the knowledge–policy process as it relates to food and transport systems. Workshop contributed to participants’ (1) abilities to engage with multisectoral stakeholders, (2) construct a shared language and understanding of urban challenges, (3) improve understanding of the interconnectedness across food and transportation systems, (4) facilitate dialogue across sectors, and (5) apply a systems thinking approach within their sector and professional context. Participants continued to draw on the tools developed during the workshop, and to apply systems thinking to their research and policy-making activities.

**Conclusions:**

CBSD may offer valuable opportunities to connect the research sector to the policy-making process. This possibility may contribute to knowledge to policy translation in the interconnection between the urban context, food and transportation systems, and health.

**Supplementary information:**

**Supplementary information** accompanies this paper at 10.1186/s12961-020-00663-0.

## Background

Knowledge of the connections between urban environments and health outcomes can guide the development of policies that improve urban health and health equity and promote environmental sustainability [1]. While research supports the use of systems science approaches to shed light on those complex connections [2], policy-making and implementation processes themselves are complex. Effective engagement, communication, and collaboration between researchers and policy-makers is often lacking [3, 4]. Multiple barriers to knowledge translation exist within both the research and policy sectors, and relate to the ways research is conducted, the way results are presented and disseminated (or not), and associated engagement activities with policy-makers and other actors [5–7].

Successful knowledge to policy translation implies the application of knowledge or evidence within policy-making processes and results in the creation of evidence-based and context-appropriate policies that respond to societal challenges [7, 8]. Urban planning and decision-making require a wide range of knowledge and collaboration between and across sectors relevant to urban systems [9–11]. Stakeholders from different sectors both within and outside the health sector must be engaged to effectively consider the practical issues with the implementation and evaluation of any policy. Moreover, the stakeholder engagement process should facilitate the development of shared language to describe the problem and the policy, and a shared understanding of how the policy implementation relates to distinct stakeholder incentives and goals [7].

There are many ways to support effective knowledge to policy translation [12], and recent proposals suggest exploring the use of community-based system dynamics (CBSD) to support this process in the context of urban health [1, 13, 14]. System dynamics provides an approach to explicitly model the structure of a system from the endogenous (or feedback) view, and to develop insights about how those structures drive that system’s behaviour [15]. Applications of system dynamics to urban environments and health systems stretch back to the origins of the field, with focus on population movement, business growth, and housing [16], and illicit drug policy and human service delivery [17]. While there have long been calls for systems approaches to address complex and multi-level public health challenges [2, 13, 18–20], these calls have not been met with a commensurate wave of applications of systems approaches.

CBSD builds on the foundations of traditional system dynamics to integrate a participatory modelling approach that aims to build community capabilities to learn and address complex problems through the feedback lens of system dynamics [21]. Since the codification of the idea of CBSD, many of its applications in public health have aimed to generate insights about a particular complex problem, through the construction of a shared language and broad shared understanding of the problems stakeholders desire to change, using system dynamics modelling tools [22–24]. A number of studies have explored the impact of group model-building processes on participant mental models and planning outcomes [25–29], and recent studies have explored community and programmatic factors influencing the adoption of system dynamics approaches for health policy [30]. However, to our knowledge, no study has focused on exploring the contributions of a CBSD-specific approach to knowledge to policy translation by facilitating discussion and analysis of complex problems among participants.

Urban Health in Latin America (“Salud Urbana en América Latina”, or SALURBAL) is a multinational collaborative research project which aims to promote health equity in Latin American cities by producing actionable research for policy [1, 31]. Acknowledging the potential for CBSD applications to promote knowledge to policy translation for urban health, the multidisciplinary project team developed a series of CBSD workshops to explore the potential contributions of this approach.

In this paper, we draw on the experiences of a diverse set of professionals who attended a CBSD workshop hosted in São Paulo, Brazil. Our aim is to understand the potential of this method for narrowing the gap between knowledge produced in research environments, and policy and intervention practices related to urban health. This evaluation is based on the participants’ perspectives of the knowledge they gained, shared, and generated with others during the workshop, and how they incorporated that knowledge within their professional practice during the year that followed.

## Methods

### The São Paulo CBSD Workshop

In 2018, within the context of the SALURBAL project, three CBSD workshops were designed and facilitated by SALURBAL team members across Latin America in order to apply a complex systems approach and tools to “gain insights into the complex and interrelated drivers of health in Latin American cities, and to identify effective policy levers” [14].

All facilitation teams’ members were urban health researchers with expertise in food and transportation systems. They represented countries from across Latin America, and were all affiliated with the SALURBAL project. Facilitators participated in training with CBSD method experts, where implicit and explicit workshop objectives were aligned and original CBSD scripts [32, 33] were adapted to context-specific examples and dynamics (see Langellier et al. [14] for details).

Two workshops were facilitated in Spanish and convened attendees from multiple countries, while one workshop, the focus of this study, was facilitated in Portuguese and held in São Paulo. The São Paulo workshop convened 24 experts in food systems and transportation sectors working primarily in Brazil, with regional, national, and international influence, including “elected and administrative policy-makers, members of civil society (e.g., nonprofits), and academics” [14]. SALURBAL-affiliated institutions from across the region identified and invited individuals in order to achieve diversity and balance across diverse professional sectors and experience in food systems and transportation. The workshop facilitation team decided on the size of the workshop in accordance with the resources available (number of facilitators, space, time, etc.), and whenever an invitation was declined, another individual with a similar professional profile was invited.

The São Paulo workshop spanned 1.5 days, during which the team of facilitators led participants through CBSD group model-building activities such as “Hopes and Fears”, “Graphs Over Time”, “Causal Loop Diagramming”, “Model Synthesis”, and “Action Ideas” [14, 32, 33]. Activities were sequenced to create space for participants working in food and transportation sectors to explore together how their perspectives on system structure were shared or differed from other participants, both within and across their sectors. At the end of day 1, a causal map was created to synthesize participants’ modelling work. On day 2, using the map as a reference, participants worked in mixed-sector teams to suggest policies and negotiate, as a large group, their potential impact and challenges to implementation. These activities were adapted with local examples to introduce system dynamics modelling tools and provide opportunities for participants to apply these tools and exchange perspectives to understand the multiple interconnected factors related to their own specific professional contexts, food and transportation systems, and urban health. A full description of workshop design and development is provided by Langellier et al. [14].

After the three workshops, the synthesized models informed the construction of computational models of food and transportation patterns in Latin America and the Caribbean (LAC) (under development by Stankov et al.), a cross-impact balance analysis was conducted to assess relatedness between selected variables identified in the three synthesized models, and this assessment was conducted to evaluate participants’ perception and experience after the São Paulo workshop.

### The São Paulo workshop evaluation

Following the São Paulo CBSD workshop, the facilitation team designed the current study to assess its contributions to policy translation. A semi-structured interview guide was developed and mirrored pre-established workshop goals. The interview guide was piloted with three observers from the workshop, and then applied with a subset of workshop participants. The goal of this assessment was to elicit participants’ perception of the workshop’s influence on their thinking one year after the activity took place. Participants’ responses indicate the way the workshop may have contributed to their application of systems thinking to address urban challenges and apply relevant knowledge about urban systems to support policy-making. Analysis of these responses provides a lens through which to view the potential contributions of CBSD as a tool for facilitating the link between knowledge production and policy-making and policy implementation.

### Recruitment

Twelve months after the São Paulo CBSD workshop, the first author emailed all (*N* = 24) workshop participants to invite them to be interviewed about their participation in the workshop. Those who did not respond to the initial email were contacted again via email and phone, and attendees willing and available to participate were scheduled for an interview with the first author within a month of first follow-up contact.

### Participants

Table 1 provides information about the study participants (*n* = 18, 75% of all workshop attendees). Participants’ ages ranged from 34 to 72, with an average age of 52 (standard deviation [SD] = 11.3), and more than half were women (61%). They came from diverse sectors and included researchers and professors, community representatives, public servants, nonprofit and nongovernmental organization (NGO) advocates, and private sector actors. This diversity allowed the workshop to encompass a variety of experiences and perspectives from both the food and transportation sectors. Participants had spent an average of nearly 15 years working in their current field (with years of experience ranging from 2 to 52 years).

**Table 1:** Characteristics of the sample of SALURBAL São Paulo CBSD workshop participants interviewed in this study

Personal information	Total participants (*N* = 18)	
	M ± SD	*n* (%)
Age	52.1 ± 11.3	
Years working in field	14.8 ± 10.4	
Gender		
Female		11 (61.1)
Male		7 (38.9)
Field of work		
Food and nutrition		9 (50.0)
Transportation		9 (50.0)
Professional sector		
Scholar		7 (38.9)
Advocacy		5 (27.8)
Public		3 (16.7)
Other^a^		3 (16.7)
Prior to workshop		
Knew at least one SALURBAL team member		16 (88.9)
Knew at least one workshop participant		16 (88.9)
Was exposed to the notion of complex systems		9 (50.0)

^a^Indicates those individuals working in the third sector or international organizations

Sixteen (89%) of the participants had known a member of the SALURBAL team before participating in the workshop, and the same number of participants knew at least one other participant in the workshop before the event. Half of the participants had previously read or heard something related to complex systems approaches.

### Data collection procedures

All interviews were conducted in Portuguese by the first author via video conference, each lasting an average of 40 minutes (30 to 120 min). The wide range in interview duration was due to the open-ended nature of the interview questions, which allowed interviewees to expand upon each subject. These semi-structured interviews (see example questions in Additional file 1) opened with interviewees asking participants to provide information about their demographic and professional background, followed by questions that sought to facilitate their recall of their participation in the previous year’s workshop in order to anchor their responses to subsequent questions. To explore possible implications for knowledge translation into practice, the interview guide included questions that reflected themes relevant to the workshop’s intended outcomes and goals. Group model-building scripts were the base of the workshop components. Each script has clearly identified inputs and outputs, and the facilitation team worked collaboratively to choose scripts with outputs that mapped onto the desired outcomes of the workshop [32]. Briefly, they encompassed CBSD practice; engagement with the proposed activities; involvement with food and transportation systems; relation with other participants; sharing of knowledge and understanding of systems at stake; conceptualizing urban health; and understanding of systems thinking and its tools (for a more detailed description, see Table 2).

**Table 2:** Qualitative codes related to the sustained impact of the CBSD workshop from follow-up interviews with workshop participants (*N* = 18)

Themes and related codes	Definition	Example quotes
Stakeholder engagement		
Governance	References to the way decisions are made that impact how cities function including power relations	“You need, from the point of view of public policy, a structuring scope, but you also need to find alternatives so that the potential and local possibilities have more capacity and possibility to be implemented, because then you can meet local needs and take advantage of what you already have there in terms of capacities and resources of different natures”
Easiness to take better choices	Statements about which and how multiple factors are considered when making health individual choices easier	“It really means having access to environments, spaces, that facilitate your options. We have been working on this a lot from the perspective of health promotion, from the point of view of building these environments, which supports decisions and does not blame or hold the individual responsible for their choices” “May it be easy to live, let it be easy to get around, eat, practice the basic functions of everyday life. Anyway, I think that the healthy environment, or healthy urban environments are those that are organized in a way that they contribute to these processes and do not hinder it” “A space where the healthiest choice is the easiest choice for people”
Shared language and understanding of problem		
Broader definition of health	Descriptions of more encompassing definitions of health and well-being	“Environments that are able to contribute to the individual's integral health. For this environment to exist, there is a need, for example, to care for mental health, for the acquisition and type of food that these individuals are going to consume, a relationship with travel time, form of travel, transit mobility”
Enabling environments	References to built environment features to improve urban health	“For me the healthy environment has corners, there are open-air markets, there are places where people, pedestrians, meet. It is a landmark, an indicator that the space is healthy is that it has a square, it has possibilities of meeting others” “I consider [healthy] a city that has good sidewalks, a continuous, connected cyclepath, with people exchanging a car trip for an active trip, the air was going to be more breathable, people were going to exercise, they would be healthier” “It is not only how it is built, but the way we take ownership of it and use it, how much of it promotes a healthy life”
Interconnections		
Urban–rural connectivity	References to interdependencies between urban and rural areas impacting urban health	“So the idea that I can see transportation as a mean, due to its interaction with all other urban activities, it can be a facilitator, including an inducer of these processes of condensation and spatial sharing” “A whole connection between the food distribution network and the transport network, it's a connection that we didn't think about and started to think” “How food is taken, transported from production areas, from farms, to drainage, distribution”
Links between sectors, regions and processes	Statements about the connections across and between different levels of influence in the ecological model	“Understanding healthy environments it is to expand that the city promoting health is not only physical activity, it is not only air quality, it is also having access to healthy food, it is also in this sense urban agriculture, which is to have urban, metropolitan food, you have a healthy diet nearby”
Built environment features	Statements about how the built environment facilitates or hinders interconnections between people or between people and their needs	“That the space does not create barriers for the person to live their life well. May it be easy to move around, easy to have fun, easy to find what they seek, be it services or products (…). For me, a healthy urban space promotes meetings. It is organized in such a way that people can somehow live together (…). So for me the healthy space has corners, there is a street market, there is a place where people, pedestrians, meet. It is a milestone, an indicator that the space is healthy is that it has a square, it has possibilities of meeting”
Dialogue across sectors		
Sharing perspectives	Statements about sharing perspectives with other participants	“It was the question of time, the discussion of healthy eating has to do with time, with the screen time we lose, with the time to buy, with the time to process, with the time to tidy up, to work on the food through time” “I think that understanding the time dedicated to the purchase of food, to eating well, to producing less processed foods and to understand that this time is also the time that we lose when travelling, in urban mobility, a conflict of time dispute”
Bridging distinct perspectives for deeper understanding	References to changes or refinements to perspectives	“We had a discussion about urban spreading and densifying, which was an approach I had never thought of. I had a vision that the less dense the city was, the better, it would be nice for people to live far away, have space, be calm” “The workshop did something that it should always have, right, which is this integration of completely different audiences, who think completely different policies and how they interact, so this, as far as I have followed along the way, was a total innovation of the workshop, in linking a group that discusses urban mobility policy with a group that discusses healthy eating. At first it was difficult for us to find out what the connection was, then we found several” “We were able to find these very strong relationships between food and urban mobility, mainly because through mobility you make the consumer have access to better food. And also the relationship between the food that people have, how they eat, and the very practice of eating they choose” “The exercises, those different moments, the degree of diversity and detail that we managed to achieve, of course within the limits of an exercise, but anyway, the variety of links and solutions that we found was something extremely interesting, that I think it was a very precious thing, even more in terms of our agenda, this issue of urban supply, the issue of supply in general is a topic that is very crucial, but it is still a bit ‘gross’. Gross in the sense of the proposals we have, they are kind of classic proposals, and in the exercise we managed to come up with proposals that had a much more interesting variety in the sense of a local application, in actions that are not simple, because none of these sections they are simple, but actions very related to specific situations, so I found this very interesting, these multiple bridges that we found between food and transportation”
Use of systems thinking		
Taking ownership of the method	Ways in which systems thinking tools are (not) incorporated into professional work	“[The workshop] brought a reflection on a systemic approach for the people who were there that possibly nobody had (…) Traditionally, we tend to be one-dimensional, monothematic, and I think that this issue of bringing a perception of systemic logic for the business was nice, and that I say for myself, I would never have had it had I not been there” “I think the method was very impressive to me. If you ask me a year from now what you will remember, I will say it for sure, even if I do not internalize it in my work” “Since I left the workshop I had proposed myself to do a more systematic approach, with the method. I even talked to Leticia [one of the facilitators], then I even met her, right after the workshop, I asked her to give me a first steps manual (…) I did very little but it is something ‘on my radar’"

In their answers, participants were asked to highlight the connections with other workshop attendees, and shared insights within their own fields or between food and transportation issues that were generated during the event and the perceived incorporation of new ideas and ways of thinking within their current work. Participants were also asked to attribute these outcomes to a specific aspect of the workshop (see Langellier et al. [14] for details). Each interview was audio-recorded. The interview consisted of survey items (demographic questions and Likert scale) and open-ended questions. Survey items were used to present descriptive statistics of the sample, and open-ended questions were transcribed for subsequent analysis.

### Protection of human subjects

Study recruitment, data collection and analysis procedures were reviewed and approved by the Institutional Review Board (IRB) of the Federal University of Minas Gerais (UFMG). During the workshop, participants signed a consent form to participate in the project and subsequent activities as required by the IRB. For the interview, they gave their verbal consent to record and use the interview material, which was audio-recorded.

### Data analysis

We applied the framework method [34] for its applicability in multidisciplinary health research teams, enhancing objectivity in content analysis. It suggests a path to identify codes and categories, cluster them around themes, and produce descriptive conclusions. To do so, we used a combination of deductive and inductive approaches to allow the integration of existing conceptual possibilities related to CBSD evaluation and knowledge to policy translation, and the identification of emergent constructs related to urban health research and practice, that arose during the workshop.

In the deductive approach, themes and codes are pre-selected based on previous literature, previous theories, or the specifics of the research question, whereas in the inductive approach, themes are generated from the data through open (unrestricted) coding, followed by refinement of themes. In many cases, a combined approach is appropriate when the project has some specific issues to explore, but also aims to leave space to discover other, unexpected aspects of the participants’ experience or the way they assign meaning to phenomena [34].

Analysis involved five members of the author team, with the first and sixth authors reading and assigning descriptive codes to each interview transcript based on previous literature (deductive step). These two coders discussed, clustered, and refined their codes based on what emerged from the data, beyond the theoretical expectations (inductive step). The first author and a third coder (fifth author) then applied the final list of codes to the data and used matrices to visualize the coded themes across interviews, reviewing examples (quotes) from each theme. The second and third author then identified five overarching themes of the multiple coded themes. Microsoft Word and Excel (2020) were used for line-by-line coding, organization of coded text, and the creation of cross-participant thematic matrices. Quotes will be presented here anonymously, linked only to the professional background of the interviewee. Each quote has is identified with respect to a certain pattern: sector + professional engagement—for example “food systems scholar”, “food systems advocacy professional”, “transport systems advocacy professional”, “transport public manager”, and other possible combinations.

## Results

Our qualitative analysis revealed five major themes in participants’ responses that indicate how the workshop experience influenced their understanding of food and transport systems and their ability to incorporate this understanding into practice to support policy-making processes: stakeholder engagement, shared language and understanding of the problem, interconnections, dialogue across sectors, and use of systems thinking (see Table 2). In this section, we provide summaries for each theme and complement these summaries with direct quotes from participants (see other relevant quotes in Table 2).

### Stakeholder engagement

The workshop was organized and facilitated by a team of academic researchers; however, participants from across professional stakeholder groups also reported that they felt they could express themselves freely and openly. Modelling activities were central to building and maintaining the participants’ engagement with each other. One participant remarked on the evolution of the group over the one-and-a-half-day workshop:

*If you think about the duration of a workshop and the diversity of participants, I think that a certain miracle was achieved, in the sense that there were people who came from very different fields, different practices, etc., and when you think about the degree of non-connection that existed at the beginning and [the degree of connection] at the end, I think there was a very important evolution (…) everyone was super interested in looking into the other people’s ideas and integrating them with their own, in a positive way, influencing these ideas to include their elements.* (Food systems scholar)

Participants also shared that by mapping the links between their system and the other (food or transportation), they could “find a broader set of political allies” from different professional backgrounds and sectors. Moreover, both plenary activities moderated by the facilitators and small group modelling work seem to have levelled power dynamics across participants, and attendees noted the acknowledgement of their ideas within the jointly built causal loop diagrams and a resulting sense of ownership of the results.

The workshops appeared to be successful in engaging a diverse group of sectors from various professional and educational backgrounds and building trust among these actors. Many workshop participants reported being previously acquainted with workshop organizers and/or other participants (61% and 89%, respectively); the workshop strengthened these existing relationships and facilitated the building of new connections.

### Shared language and understanding of the problem

A number of participants described how the workshop facilitated the development of a more comprehensive and shared understanding of issues related to urban health. This result seemed to relate to the construction of a shared language around the issues at stake, mainly linked to transport and food systems. For example, as participants worked in small groups, they needed to negotiate the language and meanings of the variables they included in their causal maps. Some participants pointed out how the modelling activities provided tools to lay bare the current goals of public policies in urban areas. This would, according to participants’ statements, make it easier to thoughtfully interrogate those goals and in turn provide the opportunity to shift focus and introduce new ideas. For example, it became clear in several diagrams that the goal to reduce travel time had promoted car use, an issue at the core of many public policies (see the full diagram in Langellier et al. [14]), and this developed as a critical debate throughout the workshop. Participants also remarked that the workshop broadened the targets of the policies they sought to influence or implement as they were prompted to consider factors related to the other sector. One participant elaborated on this expanded conceptualization of health:

*Environments that are able to contribute to the individual's full health—for this environment to exist, there is a need, for example, for mental health care, for concern regarding the acquisition and type of food that these individuals are going to consume, for a consideration of travel time, means of transportation and commuting patterns, transit mobility.* (Food systems advocacy professional)

The majority of the participants interviewed described how their thinking about the problems facing food and transportation systems were broadened and refined through modelling with other attendees. Another participant reflected on how their understanding of the issues became simultaneously more complex and yet more in sync with others:

*"It is productive to be working with a different group of people, to be expanding the network, to meet people who have a perspective aligned with yours about another world, or have another vision so that you can align yours with theirs. In that way, it was very good."* (Food systems scholar)

The core concepts, temporal and spatial scales, local interactions, connectivity, and relevant policies and interventions related to transport and food systems were examined and dissected by a group of actors with diverse experience and perspectives. Throughout the workshop, the construction of causal loop diagrams allowed for the identification of feedback loops between variables and of potential windows of opportunity for interventions and policies. The concept of a healthy urban environment served as a grounding and motivating framework for a shared perspective, inviting participants to reflect upon what an ideal environment would look like and what characteristics it would have.

### Interconnections

An important impact of participating in a CBSD workshop is the realization that perceptions that were previously understood to be common sense were overly simplistic, or constrained within one domain or field. The process of building causal loop diagrams offered the possibility of enlarging horizons and adding complexity to previously held beliefs, bringing to light interconnections between whole systems or within them, between their inner elements.

Some of the participants described having recognized connections between food and transportation systems that relate to rural and urban environments, acknowledging the complex network that is required to move food in our hegemonic food system from where it is produced to where it is sold or consumed:

*"We were able to find these very strong relationships between food and urban mobility, especially because through mobility you make the consumer have access to better food. And also the relationship between the food that people have, how they eat, and even the actual way of eating they choose."* (Transport systems advocacy professional)

While the workshop was designed to explore connections between food and transportation systems, in the process participants explored and uncovered feedback loops linking individuals and their behaviours to the environment, cultural norms, time, and multiple interdependencies between urban and rural contexts. The urban environment was therefore highlighted as a place that should facilitate interconnections between people, services, opportunities, recognizing that the way it succeeds (or fails) plays a role in determining health.

*That the space does not create barriers for the person to live their life well. May it be easy to move around, easy to have fun, easy to find what they seek, be it services or products (…). For me, a healthy urban space promotes meetings. It is organized in such a way that people can somehow live together (…). So, for me, the healthy space has corners, there is a street market, there is a place where people, pedestrians, meet. It is a milestone, an indicator that the space is healthy is that it has a square, it has possibilities of meeting.* (Food systems scholar)

Workshop structure allowed a diversity of themes and subsystems related to food and/or transport systems and urban environments to be discussed. One of the loops that explicitly made the connection between food and transportation systems described time as an important interrelated variable: “the more time available (not spent on motorized transport) people have, the more time they can use to prepare food in their homes, and the more time they spend in preparing food, the less time available they will have” (extracted from the workshop report). Another contribution of the interconnections discussions was the surfacing of urban agriculture and other options for shortening transportation chains from food production to consumption.

### Dialogue across sectors

A majority of interviewees (*n* = 12 or 67%) remarked that the group model-building activities, where stakeholders worked between food and transportation sectors to build the causal loop diagrams, produced the most striking and memorable workshop moments. Participants reflected on the discussions and insights that emerged over the course of the workshop, highlighting the importance of the small group activities interspersed with plenary sessions. The opportunity to share and engage in open dialogue was observed within and across sectors. This reflects the potential of the CBSD approach to strengthen connections between the research community and public servants and policy-makers, and to link professionals across different areas.

*As far as I have followed along the way, was a total innovation of the workshop, in linking a group that discusses urban mobility policy with a group that discusses healthy eating. At first it was difficult for us to find out what the connection was, then we found several.* (Food systems public manager)

One participant from a transportation-focused NGO discussed the shift from working on modelling within their own sector to working with attendees from the food sector:

*We managed to get the group out of the commonplace of the transportation issue. [People from the food sector] brought a different view, they were fascinated with what we were discussing in our group and (…) we were quickly fascinated with the other's issue. And I had the chance to talk about my perspective and they liked it very much. [They said], “Wow, I didn't know that!” and then there was an alignment, there was a shared perspective.* (Transport systems NGO professional)

Interestingly, several participants noted that the process was not free of conflicts or differing perspectives. On the contrary, conflicts surfaced quickly as attendees were forced to be very explicit in naming and describing the relationships between variables within their models using system dynamics diagramming conventions. In one participant’s words:

*[It is] a methodology that has a larger capacity to make conflicts emerge, even in a group that was not very confrontational. But when we got down to the relational detail of the elements that were appearing in the matrix, evidently conflicts started to arise. That was good."* (Transport systems scholar)

The CBSD workshop provided a space for participants to expand understanding of their “home” sector, to gain knowledge about another sector’s functioning, to develop insights that were unique to the gathering of that specific group, and to identify multiple, complex system connections and dynamics between food and transport systems.

### Use of systems thinking

The CBSD approach itself was described as reflective and impactful by many participants, who highlighted that they continue to draw on these concepts in their work. In terms of the approach’s application for exploring a particular problem or the effects of a policy, one participant highlighted:

*[the] importance for us to follow a reflective process and the identification of realities that really deepens the different layers of the relationships between situations, themes, realities. That makes us much more capable of understanding and identifying both challenges and potential.* (Food systems scholar)

Participants described integrating some of the systems thinking concepts within their work, and in particular concepts surrounding the feedback loops discussed. One participant who works on sustainability and transportation issues in public policy remarked:

*Perhaps the workshop pointed out ways, what are the relevant themes to discuss the issue of urban and city, also think in the perspective of food and nutritional security, healthy food, this was used in some way in these discussions with the technical teams.* (Transport system public manager)

While modelling was still seen as a challenge for the number and complexity of variables and the various relationships between them (positive, negative, stocks and flows, feedback of reinforcing or balancing loops [35]), participants shared several other topics which might benefit from a similar approach. One noted that while system dynamics capabilities are developed over time, even an imperfect application can be useful:

*It was not easy to understand that method. If I go to another workshop, I will make a lot of mistakes, but the method uses a different way of approaching all the issues, and not drowning in the complexity of the problems.* (Transport systems advocacy professional)

Despite identifying the challenges related to complex systems ideas and CBSD, workshop participants appeared to have internalized and applied the tools obtained in the workshop throughout the year following the activity. The workshop provided participants with a theoretical and practical introduction to systems thinking and an approach for organizing understandings about systems within multiple sectors.

## Discussion

Effective urban planning and policy-making for urban health require an applied understanding of the complex and interconnected systems that include urban environments, as well as the collaboration of actors within and across sectors. A significant body of work has focused on strategies for improving the translation of knowledge about urban systems into policy. Nevertheless, the specific pathways and characteristics that determine the success of knowledge to policy translation for urban health are not completely understood, and the process continues to face many challenges [8]. These obstacles can relate to issues of competing ideas or incentives, and a lack of engagement of stakeholders across sectors; communication failures, including issues of format, focus, and timing; a lack of structures or mechanisms for connecting the research and policy sectors; and limited capacities of local policy actors to interpret and apply existing knowledge [3, 4].

Drawing on the perspectives of CBSD workshop attendees, we find support for CBSD as a potential approach for overcoming some of these challenges and improving knowledge-to-policy translation for urban health, for its capacity to promote transdisciplinary and intersectoral exchange and engagement that in traditional urban planning practice is not always doable for the limitations pointed above. Participants indicated that the workshop contributed to their understanding of and ability to apply relevant knowledge to urban policy-making processes in multiple ways. Each of the five general themes surrounding this impact that emerged from our qualitative analysis highlight important concepts for promoting effective knowledge translation: stakeholder engagement, shared understanding of the problem, perception of interconnections, cross-sector dialogue, and use of systems thinking.

### On stakeholder engagement

The “high-touch, low-tech” markers and chart paper approach to modelling was accessible and stimulating, which aligns with other observations of CBSD in the literature [23, 36, 37]. In contrast to a facilitator-led modelling process, where the onus of reflecting participants’ ideas and resolving differing perspectives falls on the facilitator, a small team modelling-based process created opportunities for participants to “hold the pen”, practicing and negotiating conflicts themselves and enhancing engagement and ownership of the process and method. It has been noted by other authors that group model-building creates an environment where conflicting perspectives can interact and make it possible for creativity and innovative ideas and insights to arise [14, 38].

### On shared language and understanding

The workshop promoted multiple interactions among participants and allowed reflections that emerged and converged toward common understanding around priorities and challenges. Participants’ reflections suggest that workshop activities created space to express and exchange perspectives, identify intersections and overlap, and promote a more holistic view and conceptualization of urban health. The potential of group model-building and causal loop diagramming to help create and/or deepen shared language and understanding of a problem has been noted before by other authors and highlighted as an important feature to orient consensus for action [38–42].

### On interconnections

Understanding the interconnectedness of systems such as food and transportation in an urban environment is vital to achieving more comprehensive, holistic, and effective approaches to research, knowledge production, translation, and practice [5, 35]. To do so, it is necessary to acknowledge that there are no “side effects” of urban policies or interventions—only effects that were not previously understood [43]. Building cities to support healthier food and transportation systems for healthier individuals and communities requires incorporating an understanding of interconnections between those systems. The CBSD workshop assessed in this study appears to have contributed to participants’ understanding of complex food and transportation systems, offering tools and insights to approach their interconnections and the potential impacts of policies and other interventions.

### On dialogue across sectors

Designed to promote interactions across professional sectors and roles, the workshop built trust among participants and supported the robust relationships necessary for effective evidence-based policy-making processes. The use of qualitative and participatory methodologies such as CBSD can support knowledge to policy translation by promoting interaction between perspectives of diverse social actors involved in different sectors and with distinct—yet interconnected—responsibilities and roles in society [44]. Effective translation of knowledge within and between different systems requires a robust understanding of each system [45]. An understanding of these dynamics in turn supports urban planning and urban policy-making, implementation, and evaluation processes.

### On use of systems thinking

Urban systems are complex and nonlinear, composed of multiple ongoing interactions and dynamics. They are considered to be systems that learn, adapt, and evolve [44]. A notable characteristic of CBSD methodology, and more generally participatory system dynamics modelling and group model-building approaches, relates to the potential for a learning process that can extend beyond the original workshop’s location and duration [46]. The application of participants’ broadened understanding of the complexity and links between the infinite variables within a complex urban system may support more effective urban policies and interventions that take into account these connections. A widespread shift to systems thinking, if applied to practice, has potentially enormous implications for policy-making processes.

Our interviewees provided criticism and suggestions, and noted difficulties faced during the workshop. Most of these related to issues regarding implementation of the ideas and insights in “real life”, especially in terms of power relations and bureaucracy, workshop duration, or participants’ representativeness. It was not within the scope of this work to discuss these issues, as CBSD methodology considers these limitations, and they were openly discussed with participants throughout the workshop and interviews. Many factors are known to limit the ability of policy-makers to apply existing and emerging knowledge about health impacts on urban planning and decision-making processes [4]. We believe that CBSD workshops present a valuable approach to addressing some of these limiting factors, with the potential for expanded implementation for urban health and other complex systems and problems.

Complex system approaches have the potential to inform public policies and help understand their effects, their effectiveness, and their direct and indirect costs [44]. CBSD builds engagement and trust, and creates space for the co-construction of new ideas. This approach can strengthen the research to policy linkages required for the effective application of existing knowledge for policy design, implementation, and evaluation, helping to overcome traditional urban planning obstacles.

The way cities are built, organized, and managed has important implications for well-being, health, and environmental sustainability. Urban spaces are places where a high density of people, institutions, and economies interact and engage in complex, productive, and innovative ways [47, 48]. Planning for, executing, and evaluating urban policies must therefore involve and integrate the dynamic participation of community members, research institutions, and government actors. Achieving effective integration across sectors requires the establishment of a shared language to facilitate communication and promote reflection surrounding the cross-cutting nature of urban issues.

Policy analysis undertaken with a perspective that integrates the knowledge and experience of multiple sectors, scales, and actors promotes a more accurate inclusion and assessment of externalities, vested interests, and points of view. Complex systems approaches to understanding urban dynamics permit an integrated observation of urban environments, across scales (from microanalysis to macroanalysis), sectors, and perspectives [44].

CBSD and systems thinking therefore emerge as practical and theoretical approaches to addressing the gap between knowledge production and policy-making processes for urban health. These methods can help address complex problems within complex systems, supporting urban planners and decision-makers in disentangling variables and distinguishing “complex” from “complicated”. Evidencing systems’ variables and their interconnection, together with feedback loops of reinforcement and/or balance, fosters stakeholder understanding of health inequities related to systems such as food and transportation. Recognizing disparities in access to resources within and across systems may strengthen engagement and orient urban planning toward interventions to build better equity in urban health. The workshop experience described here focused on food and transport systems in a Latin American urban environment. Similar approaches offer the potential to support understanding of multiple sectors and of action required across sectors—for example, for more effectively managing natural resources and building more sustainable cities that promote both human and planetary health.

## Limitations

The current study presents a number of limitations.

Despite efforts to recruit and interview all 24 workshop attendees, only 75% were available and willing to participate in a follow-up interview. It is possible that those who responded were those who found the workshop particularly useful or impactful. Additionally, although the diversity of participants’ backgrounds represents an important feature of this study, our goal was not to analyse the relationship between participants’ demographic or professional backgrounds and their workshop experience. The process of developing causal loop diagrams and feedback loops were conducted in groups, by design. Therefore, it is not possible to make an analysis based on individual contributions. As this is a qualitative approach with a small number of subjects, by design it is not possible to evaluate the representativeness of participants’ sectors. Rather than highlight individual contributions or representativeness of participants and groups, our objective was not to be exhaustive on how the workshop can have objective impacts in participants’ lives but to observe the experiences of participants as a whole and the complexity and creativity that could arise from this activity.

Interview questions about the continued use of systems thinking tools and the participants’ current work were also self-reported, and as such are potentially unreliable indicators of actual use. While the interviews were conducted 12 months after the workshop, in an effort to reduce potential positive bias from the immediate enthusiasm produced at the workshop, this delay may itself have caused a high recall bias by differences in the accuracy or completeness of the recollections retrieved. We also recognize missed data collection opportunities that would have enriched our data analysis, such as pre-workshop and immediately post-workshop surveys or interviews.

One advantage of community-based research techniques is the capacity to engage diverse perspectives to achieve an improved understanding of complex systems and the forces driving health disparities [49]. Future CBSD workshops might orient the selection of participants based on observed health disparities in Latin American urban contexts in order to create space for discussions of the factors and processes behind these disparities, as well as potential solutions to mitigate inequities.

Finally, it was beyond the scope of this study to empirically assess changes to the policy or research processes related to food and transport systems in Brazil as a result of the workshop. Nor did we attempt to observe the workshop’s impact on the development or implementation of specific policies or interventions. Such an assessment, if feasible, would require a longer-term monitoring process involving ongoing policy surveillance, in addition to systematic and sustained follow-up with individual participants. Future efforts could focus on identifying key moments for urban policy and program development in order to orient the implementation of CBSD workshops for urban planners and policy-makers involved in these processes.

## Conclusions

The use of systems science modelling approaches in public health is often introduced in the context of research. This study presents initial support for further exploration of the use of a participatory approach to systems science—CBSD—for a different purpose: narrowing the gap between scientific knowledge and real-world policies and their implementation. The CBSD workshop attendees we interviewed indicated that both the systems thinking tools introduced and the process of participatory modelling allowed them to generate a shared understanding of a complex issue and create new relationships with others, besides creating space for disagreement and constructive dialogue.

Additionally, this work demonstrates an approach to evaluating CBSD applications with diverse stakeholders, and provides additional support for urban health as a unifying framework for understanding complex urban environments as they relate to transportation and food systems. The tools and methodology developed may be of interest and use for other CBSD initiatives and contexts. Their strategic and effective application may serve to bring together researchers and policy-makers from across sectors, support dialogue and mutual learning, and promote systems thinking about effective policies and interventions to improve urban health, equity, and sustainability.

## Consent for publication

Not applicable.

## Competing interests

The authors declare that they have no competing interests.

## Supplementary information

**Additional file 1:** Questionnaire.

## Data Availability

The interview script and additional supporting quotes extracted from interview transcripts are included as supplementary material. Additional clarification and supporting materials may be made available upon request to the corresponding author.

## Abbreviations

CBSD: Community-based system dynamics
SALURBAL: Salud Urbana en América Latina (“Urban Health in Latin America”)
NGOs: Nongovernmental organizations
IRB: Institutional Review Board
UFMG: Federal University of Minas Gerais
CNPq: National Council for Scientific and Technological Development (in Brazil)
