# Supplementary material for: Promoting knowledge to policy translation for urban health using community-based system dynamics in Brazil
Source: Health Res Policy Syst. 2021 Apr 1;19:53. doi: 10.1186/s12961-020-00663-0 (PMC8015032; doi:10.1186/s12961-020-00663-0)
Supplement: Supplementary file 1 — Additional file 1: Questionnaire. [file 12961_2020_663_MOESM1_ESM.docx]

# Additional File A

| **Original Interview Script** |
| --- |
| Can you tell me your name (1)? |
| and date of birth (2)? |
| (3) What is your field of action? (transport / food) How do you act? (university, government, advocacy, etc… ) |
| (4) Time in this function: |
| (5) Have you changed your field of activity in the past year? |
| i. How did you get the invitation to the workshop? (Someone you knew, indication, others) |
| ii. Did you go to Sao Paulo just to attend the workshop? Did you participate fully in the activities? If not, in which part you were not present? |
| iii. From what you remember of the activities developed during the workshop, what were the most striking moments for you? |
| 1.a. Did you already know any of the other workshop participants? What about the Salurbal project team? how (collaboration, know of them, have read their work)? |
| 1.b. After the workshop, did you have any contact with the other participants? How / in what situation (people you met in the workshop - did not know before)? Propositional / occasional? What about the Salurbal project team? Were any of these encounters due to the meeting in the workshop? |
| 1.c. If so, what was the purpose of these contacts? Did any of those contacts result in new activities, collaborations? |
| 1.d. If not, why? |
| 1.e. In addition to the proposed themes (transport and food), did the workshop have developments for other topics? |
| 1.f. Did participating in the workshop influence your life in other settings, positively or negatively? |
| 2.a. Have you had experience with systems thinking / systems thinking / complex systems before the workshop? |
| 2.b. If yes, in what context? (workshop / workshop, readings (book, blog, formal training, etc.) |
| 2.c. Have you had prior experience with any of the methodologies developed during the workshop (eg, group building of models, causal link diagrams, expectations and fears, graphics over time)? |
| 2.d. If yes, in what context? |
| 2.e. On those occasions how was your participation? (watched, moderated ...)? |
| 3.a.1.Did you feel that you were able to express your perspectives during the workshops? Did you feel contemplated, or could you see incorporated your perspectives in the model(s) (intermediary and synthesis model)? |
| 3.a.2. Do you think during the workshop the participants' opinions tended to converge or diverge? Do you identify with this alignment / detachment? |
| 3.b. Would you like to participate again in a similar workshop? What topics would be interesting for a workshop with a similar design? |
| 3.c. Would you recommend this type of workshop to a colleague? Would this colleague have a specific profile? |
| 3.d. Do you think that among the participants of our workshop lacked any type of profile? |
| 3.e. Do you consider that a workshop with similar design would be useful / interesting within your professional context? why? |
| 4.a. What was the contribution of the workshop in your understanding of “healthy urban environments”? |
| 4.b. What was the contribution of the workshop to your understanding of the relationship between food and transportation systems? |
| 5.a. What do “healthy urban environments” mean to you? |
| 5.b. 1. Under your perspective, what strategies or types of strategies could be undertaken to create more healthy food /transport (one option) environments? |
| 5.b. 2. Did your understanding of the subject or the strategies you listed change as a result of the workshop? If so, how? (Listen for responses in the following types, framing/confirming understanding): |
| 5.b. 3. Has any part of the workshop had any major influence on this change? |
| 5.c. In your opinion, what is the contribution of the workshop as a whole? (the ‘general message’) |
| 6.a. Did any of the methodologies used influence your PERSONAL professional work? If yes, how? And the discussions and results of the workshop? |
| 6.b. Has this personal influence generated repercussions in your professional context? |
| 6.c. (if YES) Have any interventions / program / project / policy or other objective outcomes been influenced / revised / planned / developed after these changes?? |
| 7.a. (Period effect) In this last year, since we did the workshop, was there any important milestone, or most striking points in your professional life? |
| 7.b. Was the feedback received after the workshop enough (Report submitted, LAC register invitation, CIB questionnaire ...)? Does this interview contribute in any way? Any suggestions for feedback strategies after the workshop? Or of how the project could keep in touch with the participants? |
| 7.c. Would you add / change anything in the workshop? |
| 7.d. Do you have anything else you would like to add? |
